# Supplementary figures and images for: Epidemiology of intestinal helminthiasis with an emphasis on taeniasis in Chipata district of the Eastern province of Zambia
Source: PLoS Negl Trop Dis. 2023 Nov 20;17(11):e0011561. doi: 10.1371/journal.pntd.0011561 (PMC10695371; doi:10.1371/journal.pntd.0011561)

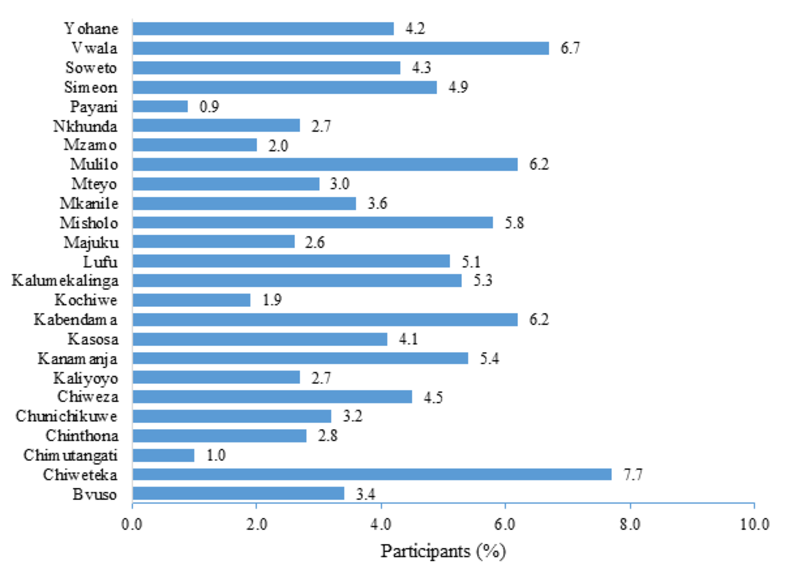

Supplement: S1 Fig — (TIF) [file pntd.0011561.s001.tif]
