# Supplementary material for: Epidemiology of intestinal helminthiasis with an emphasis on taeniasis in Chipata district of the Eastern province of Zambia
Source: PLoS Negl Trop Dis. 2023 Nov 20;17(11):e0011561. doi: 10.1371/journal.pntd.0011561 (PMC10695371; doi:10.1371/journal.pntd.0011561)
Supplement: S1 Table — (DOCX) [file pntd.0011561.s002.docx]

**S1 Table. Predictors of a positive taeniasis result on copro Ag-ELISA.**

|  | Predictor variable | OR | 95% CI | *p* value. |
| --- | --- | --- | --- | --- |
| Village | Bvuso | 0.0 | 0.0 | 0.997 |
|  | Chiweteka | 6.9 | 2.1 – 23.1 | 0.002 |
|  | Chimutangati | 0.0 | 0.0 | 0.998 |
|  | Chinthona | 0.0 | 0.0 | 0.997 |
|  | Chunichikuwe | 0.0 | 0.0 | 0.997 |
|  | Chiweza | 0.9 | 0.2 – 4.6 | 0.918 |
|  | Kaliyoyo | 1.0 | 0.2 – 6.3 | 0.981 |
|  | Kanamanja | 7.3 | 2.1 – 24.9 | 0.002 |
|  | Kasosa | 4.9 | 1.4 – 17.7 | 0.015 |
|  | Kabendama | 7.0 | 2.1 – 23.7 | 0.002 |
|  | Kochiwe | 0.0 | 0.0 | 0.997 |
|  | Kalumekalinga | 3.7 | 1.0 – 13.2 | 0.046 |
|  | Lufu | 2.9 | 0.8 – 10.6 | 0.118 |
|  | Majuku | 3.4 | 0.8 – 14.2 | 0.088 |
|  | Misholo | 13.4 | 4.0 – 44.5 | <0.001 |
|  | Mkanile | 2.9 | 0.7 – 11.4 | 0.134 |
|  | Mteyo | 4.0 | 1.0 – 15.7 | 0.044 |
|  | Mulilo | 0.0 | 0.0 | 0.995 |
|  | Mzamo | 0.0 | 0.0 | 0.997 |
|  | Nkhunda | 5.8 | 1.5 – 22.0 | 0.009 |
|  | Payani | 0.0 | 0.0 | 0.998 |
|  | Simeon | 0.6 | 0.1 – 3.4 | 0.531 |
|  | Soweto | 2.0 | 0.5 – 8.2 | 0.335 |
|  | Vwala | 4.8 | 1.4 – 16.4 | 0.013 |
|  | Yohane | Ref |  |  |

OR, odds ratio; Ref, reference standard.
